# Implications and Assessment of the Elastic Behavior of Lamins in Laminopathies

**DOI:** 10.3390/cells5040037

**Published:** 2016-10-14

**Authors:** Subarna Dutta, Maitree Bhattacharyya, Kaushik Sengupta

**Affiliations:** 1 Biophysics & Structural Genomics Division, Saha Institute of Nuclear Physics, 1/AF Bidhannagar, Kolkata 700064, West Bengal, India; suborna.dutta@gmail.com; 2 Department of Biochemistry, University of Calcutta, 35, Ballygunge Circular Road, Kolkata 700019, West Bengal, India; bmaitree@gmail.com

**Keywords:** lamins, mechanotransduction, elasticity, single molecule force spectroscopy

## Abstract

Lamins are mechanosensitive and elastic components of the nuclear lamina that respond to external mechanical cues by altering gene regulation in a feedback mechanism. Numerous mutations in A-type lamins cause a plethora of diverse diseases collectively termed as laminopathies, the majority of which are characterized by irregularly shaped, fragile, and plastic nuclei. These nuclei are challenged to normal mechanotransduction and lead to disease phenotypes. Here, we review our current understanding of the nucleocytoskeleton coupling in mechanotransduction mediated by lamins. We also present an up-to-date understanding of the methods used to determine laminar elasticity both at the bulk and single molecule level.

## 1. Introduction

The nucleus of a eukaryotic cell plays a deterministic role in cellular fate and homeostasis. It is a double membrane bound organelle comprising of an inner nuclear membrane (INM), an outer nuclear membrane (ONM), and a nuclear pore complex (NPC) and confines the tightly packed chromatin. A dense meshwork of proteins underneath the INM imparts proper structure, shape, and rigidity to the nuclear membrane and acts as sites of attachment for NPCs and chromosome tethering [1,2]. Lamin proteins are type V intermediate filament proteins and are principal components of nuclear lamina. There has been an upsurge of interest in lamin biology since the discovery of an increasing number of mutations associated with lamin A, leading to at least 14 different diseases collectively referred to as laminopathies (http://www.umd.be/LMNA/). Insights into different laminopathies and the roles of lamins thereof necessitate revisiting the elastic properties of nuclear lamina. This review focuses on the structural perturbations of laminopathic nuclei associated with the mutations of lamin A corresponding to changes in viscoelastic behavior. We have presented an up-to-date status of our understanding of the role of lamins in modulating the elastic behavior of the nucleus.

## 2. Structure and Properties of Lamins

Lamin proteins were first isolated from rat liver and chicken erythrocyte nuclei as components of nuclear lamina [2,3] and later classified as type V intermediate filament proteins [4,5,6]. Lamin proteins are found in all metazoan cells with the sole exception of plants and unicelular organisms [7,8]. Based on sequence homologies, lamins can be classified into A- and B-types. In mammals, two A-type lamins (lamin A and C) are derived from the LMNA gene located on chromosome 1, whereas B-type lamins (lamin B1 and B2) are derived from LMNB1 & LMNB2 on chromosomes 5 and 19, respectively [9,10]. Other minor isoforms like lamin A∆10 [11] and germ line-specific Lamin C2 [12] are obtained from LMNA, and B3 is obtained from LMNB2 [13]. All lamins carry a tripartite structure consisting of a long central alpha helical rod domain, which are flanked by N terminal head and C terminal tail domains. The C terminal tail domain is grossly unstructured and contains a nuclear localization signal NLS [14] followed by the immunoglobulin fold (Ig-fold) [15,16] while terminating in a CAAX box that is necessary for farnesylation [17] Interestingly, B-type lamins are permanently farnesylated, whereas A-type lamins do not retain the farnesyl group.

Lamins have a propensity to self assemble into higher order oligomeric structures that are initiated by a dimer formation and propagated by parallel in-register associations at the molecular level [8,18,19]. It is now established that the head, rod, and tail domains play active roles in lamin assembly [18,20]. Inside the nucleus, A- and B-type lamins form separate networks which interact amongst themselves [21] However, the means by which individual lamins in a cell polymerize to render the final laminar structure is still shrouded in mystery. Near orthogonal meshwork of lamin fibers were visualized in Xenopus oocytes [4], while mammalian nuclei almost always exhibit irregular filamentous networks [22,23]. Recent studies on lamin assembly in xenopus oocytes have shown a near orthogonal regular network of B-type lamins, superposed by irregular bundles of A-type lamins [24]. Therefore, it can be conjectured that B-type lamins, especially B1, form the plinth on which the laminar superstructure is assembled. This view finds support from the knockdown experiments of lamin B1, which culminates into an increased mesh size of lamin A/C and B2 with an additional formation of nuclear blebs [21]. The lamin proteins form an elastic component of the lamina and act as “shock absorbers” [25,26], which therefore regulate the mechanical behavior of the nucleus. It is now established that viscoelasticity of the nucleus depends entirely on A-type lamins, while B-type lamins resist nuclear deformation in response to force [27].

## 3. Expression of Nuclear Lamins during Development and Differentiation

A- and B-type lamins not only differ in their structural features but also in their expression patterns as well. B-type lamins are expressed as the first intermediate filament protein of the nucleus in most embryonic stem cell types, except in one instance where lamin B was shown to be superfluous in differentiation [28], while lamin A sets in during differentiation, although the expression patterns varies among animal to animal [29]. Xenopous oocytes contain LB3 as the principal, whereas minor forms LB1 and LB2 express at the time of blastula transition and gastrulation [30,31,32]. Drosophila and chicken embryos exhibit a similar trend of lamin expression [33,34]. In mice, lamin A/C first appear at the trophoblast stage (E.D 9), followed by expression in myoblasts and mesenchymal tissues on embryonic days 10 and 11, respectively [35]. Thus, it is only lamins B1 and B2—not lamin A/C—that are expressed in undifferentiated mouse and human embryonic stem cells [36]. B-type lamins are very essential during animal development. Site-directed mutations on B-type lamins of drosophila cause fatality at different embryonic or late pupal stages [37,38]. Lmnb1-/- and Lmnb1-/-/Lmnb2-/- are born smaller in size and succumb to death within minutes after birth due to lung and brain developmental defects [28,39,40]. On the other hand, animals with deficiency of lamin A/C suffer from postnatal retardation of growth [41] associated with skeletal and cardiac muscle disorder [41,42]. Again, stiffness of a tissue is largely dependent on the relative expression ratio of A- and B-type lamins. Soft tissues such as the brain exhibit a low lamin A:lamin B ratio that is reversed for harder load-bearing tissues such as muscles, bone, and cartilage [43]. Correspondingly, embryonic stem cells (ESCs) expressing low lamin A/C levels exhibit higher plasticity compared to differentiated cells [44,45].

## 4. Laminopathies and Role of Lamin A in Mechanotransduction

Lamins have gained fresh impetus due to the uncovering of over 450 mutations only in lamin A, which are associated with 14 human diseases termed as laminopathies. Surprisingly, only a handful of diseases have been identified with lamin B1 and lamin B2 mutations [46], which further support the notion that B-type mutations are embryonically lethal, while A-type lamins are not. Some of the laminopathies, including dilated cardiomyopathy (DCM), Emery–Dreifuss muscular dystrophy (EDMD), Hutchinson–Gilford progeria syndrome (HGPS), and familial partial lipodystrophy (FPLD), are characterized by mechanically compromised nuclei that are often deformed and lack integrity, as observed in tissue samples from patients [47,48]. Heart muscle tissues were obtained as biopsy samples from DCM patients [49] and from mouse models [50,51] as well as DCM and EDMD models in Drosophila, where body wall muscle showed abnormally elongated nuclei with a concomitant reduction in nuclear stiffness [52,53]. Moreover, the human and murine muscle tissues bearing DCM mutations showed increased nuclear fragility and discontinuity in nuclear lamina, as visualized by electron microscopic analyses [54,55]. On the contrary, LMNA mutations cause a HGPS result in stiffened nuclei as observed in patient fibroblasts [56,57]. Thus, the mechanical model comes in the scenario that explains that sensing and response to external mechanical cues by the nucleus is mediated by nuclear lamins. According to the mechanical model, aberrant mechanical response from the nucleus due to lamin A mutations causes increased vulnerability of the nuclei of load bearing tissues encountering mechanical stress. As a consequence, the misshapen nuclei of the laminopathic cells respond poorly to stretching forces. ”Mechanosensing” occurs at the level of the extracellular matrix (ECM), which generates tensional force on the cell. This in turn is sensed and processed by the cytoskeleton as biochemical signals and mechanical forces that terminate into the nucleus, thereby eliciting effector responses [58]. The nuclear lamina is physically connected to the cytoskeleton via the linker of nucleoskeleton and cytoskeleton (LINC) complex, which plays a crucial role in mechanotransduction [59]. The LINC complex is comprised of SUN ( Sad1 & UNC-84) domain proteins spanning the INM [60] and is connected to nesprins (also referred to as Syne, Myne, and NUANCE) at the ONM [59,61,62]. Different isoforms of nesprin also bind actin and microtubules and cytoplasmic intermediate filaments [63,64]. Silencing or depleting the components of the LINC complex results in nuclear envelop deformities, thereby suggesting that these components are necessary to maintain proper shape and structure of the nucleus in response to mechanical forces [65,66]. The support for this statement comes from the observations that defective nucleocytoskeletal coupling is observed in cells expressing mutant lamin A proteins [52], thereby disrupting the proper mechanotransduction pathway. Recent studies in *C. elegans* models have shown that L535P causing EDMD perturbs the mechanical response of muscle nuclei in response to strain. As expected, the LINC complex and emerin are required to elicit this mechanical response of the nuclei [67]. This is an important addition in the field, as the experimental procedures determining nuclear strain are based on the whole living animal. This is reminiscent of the mammalian cells, which are also characterized by improper localization of various components of the LINC complex [68,69]. At the same time, the interaction of various downstream signaling molecules with lamin A might be perturbed [70]. Load-bearing tissues such as muscles, cartilage, and bones containing elevated levels of lamin A are worst affected in diseases such as EDMD, DCM, and HGPS and are characterized by aberrant nucleocytoskeletal coupling [52].

## 5. Measurement of Viscoelasticity of Lamin A Proteins

Nuclear deformations observed in different laminopathies include nuclear envelop blebbing, elongated nuclei, lamina thickening, and overall fragility of the lamina [71]. These phenotypes can result from an alteration of viscoelastic behavior of the nuclear lamina. Several biomechanical techniques such as micropipette aspiration, cell strain and compression, and atomic force microscopy have aimed to probe mechanical properties of nuclei [44,72,73], where chromatin contributions also had to be taken into consideration [25]. Similar viscoelastic properties of the actin cytoskeleton have been shown earlier via particle tracking microrheology experiments in the cytoplasm of stem cells and differentiated fibroblasts. It must be borne in mind that stem cells due to their intrinsic plasticity are assumed to be an ideal viscous system, whereas differentiated fibroblasts (as used in the study) possess both viscous and elastic components [74]. However, how the viscoelasticity of the individual proteins contribute to the bulk behavior of the cell posed an interesting problem to biophysicists and cell biologists. Likewise, the viscoelastic properties of the actin protein have been studied in great detail via rheological measurements [75,76,77]. Similar viscoelastic studies also exist for cytoplasmic intermediate filament proteins such as desmin, keratin, and vimentin [78,79,80,81,82]. Both desmin and vimentin have shown strain stiffening behavior from mechanical rheometry [83]. Pioneering studies have also focused on quantitative rheological measurements of lamin B1, where the stiffness of the matrix increased with strain. This strain hardening was hypothesized to resist forces leading to nuclear deformation prior to nuclear envelope rupture in prophase [83]. However, lamin B1-depleted cells retained normal mechanical functions despite occasional nuclear blebbings, while A-type lamin-deficient cells had attenuated nuclear stiffness [84]. Lamin A plays a pivotal role in the pathophysiology of diseases linked to muscles where mechanical force transmission is the prerequisite for their proper functioning. Therefore, the effect of the viscoelasticity of lamin A is an important avenue to explore. However, such study with lamin A was largely plaguing the field until 2013, when the viscoelasticity of human lamin A protein, along with some of its mutants, was first elucidated [27]. The hallmark of this study lies in the unique viscoelastic response of the lamin A networks deviating from strain hardening behavior of lamin B1. This is not surprising, as there is only 50% homology between human lamins A and B1, and they have been shown to possess distinct higher order associations [85]. The mutations E161K and R190W, which cause DCM in patients [86,87,88], lead to a fragile and poorly elastic matrix, which is unable to bear shear strain deformation [27]. This clearly explains why such lamin A mutations lead to abnormally elongated nuclei with concomitant changes in elasticity.

## 6. Determination of Lamin A Elasticity at Single Molecule Level

**Figure 1:**
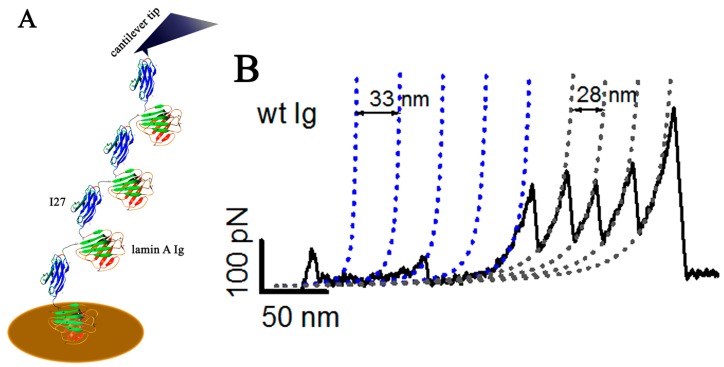
Schematic diagram of stretching of the Ig-fold domain in single molecule force spectroscopy (SMFS) and the force-extension curve. (**A**) Illustration showing the construct of alternating units of Ig and I27, which are pulled by a cantilever tip from the gold coated cover slip containing a drop of the protein; (**B**) Force extension curves plotting the unfolding force and change in contour length. The curve is fitted with a worm-like chain model of polymer elasticity. (Reproduced with permission from Bera et. al, Biochemistry, 2014).

So far, we have reviewed the bulk properties of the lamin A and how mutations affect the viscoelasticity of the lamina. However, it is of interest to elucidate how the mutations that induce secondary and tertiary structural changes at the molecular level [89] play a role in altering the stretching of the domains in response to external forces. Although earlier reports elucidating the mechanical properties of a single vimentin filament had been addressed by atomic force microscopy [90], single molecule pulling experiments on nuclear intermediate filaments using atomic force microscopy were lacking, which has been adequately addressed by Bera et al. [91,92]. Single molecule pulling experiments by atomic force microscope cantilevers of defined spring constants have been elegantly used to show a stretching of molecular domains on the application of picoNewton forces. The stretching experiments using the I27 domain of the titin giant protein enables one to plot force-extension traces by using a worm-like chain model for polymer elasticity [93] and calculate its mechanical properties such as persistence length and contour length with utmost precision [94,95]. Although it has been speculated that the uncoupling of the nucleoskeletal framework corresponds to poor force transduction from the exterior to the nuclear interior, no report has ever shown how the mutations in lamin A that cause laminopathies alter the “springiness” of the otherwise spring-like elastic protein. Recently, how lamin A protein domains unfold in response to externally applied force (Figure 1) and that response is abrogated as a consequence of mutations has been demonstrated via single molecule force spectroscopy [91,92]. In separate experiments, rods 1B and 2B, and Ig-fold domains, were pulled at an average of 1000 nm/s rate. R453W in the Ig-fold unfolded at lower force, and this data was corroborated with a steered molecular dynamic simulation where the sequential unfolding of 9 beta strands was observed. It was conclusively shown that R453W, the mutant causing AD-EDMD was intrinsically unfolded, thereby rendering the network weaker and less resilient to force [91]. Interestingly, statistics reveal that the majority of the mutations in the central alpha helical rod domain are principally concentrated in the 1B domain, and this domain possesses a mechanical resilience of up to 200% strain. This might explain why mutations in the rod 1B leads to severe phenotypes in patients afflicted with laminopathies [92]. The single molecule force spectroscopy is an excellent method to study the stretch response of a protein but is limited by the choice of the protein. As lamin proteins have a high propensity to aggregate, these are not suitable to be studied as a full-length protein. Nevertheless, it can be broken up into smaller independent fragments to generate force-extension curves, which are additive over the entire length of the protein.

## 7. Conclusions

The nuclear lamina can thus be viewed as a mechanoresponsive element of the cell where the mechanical cues are transmitted from ECM with the help of LINC complexes. Lamins being principal components are also effectors in this mechanotransduction relay by dint of its structural fluctuations and chromatin reorganization. Lamin A is of paramount importance in this relay as a large number of mutations associated with it lead to a plethora of diseases termed as laminopathies. Some of the causative factors behind these mutations are being uncovered by applying principles of physics in biology in the form of rheology and single molecule force spectroscopy. However, further investigations are underway to enable us to delineate the signaling pathways leading to the pathophysiology of laminopathies.
